# The Developmental Trajectories of Attentional Biases and Their Association With Internalising Symptoms in Children Transitioning Into Early Adolescence

**DOI:** 10.1002/ijop.70104

**Published:** 2025-08-23

**Authors:** Qiaochu Zhang, Yahui Wang, Samuel M. Y. Ho

**Affiliations:** ^1^ Department of Social Work The Chinese University of Hong Kong Hong Kong SAR China; ^2^ Department of Social and Behavioural Sciences City University of Hong Kong Hong Kong SAR China

**Keywords:** adolescents, anxiety, attentional bias, children, depression

## Abstract

Despite the developmental models proposed by Field and Lester, no studies have investigated the developmental trajectories of attentional biases and examined their association with internalising symptoms. The current study aimed to establish the developmental trajectories of self‐reported negative and positive attentional biases. Two hundred sixty four Chinese children, ranging in age from 9 to 10 years, were recruited from a primary school in Shenzhen, mainland China. Self‐reported inventories of attentional biases, active and avoidant coping styles, and internalising symptoms were completed at the first assessment by children in classrooms. After 6 months, children completed the same self‐reported inventories of attentional biases and internalising symptoms from the second to the fourth wave, with an assessment interval of 6 months. After controlling for the effect of active and avoidant coping styles, children with the trajectory membership of higher negative attentional bias were more likely to have the trajectory membership of higher internalising symptoms. Children with the trajectory membership of lower positive attentional bias were more likely to have the trajectory membership of higher internalising symptoms, after controlling for active and avoidant coping styles. The results have implications for the developmental model of attentional biases and internalising symptoms.

## 1 Introduction

### 1.1 The Development of Attentional Biases in Children

Positive or negative attentional bias is conceptualised as the cognitive tendency to selectively attend to positive or negative stimuli, respectively (Bar‐Haim et al. 2007). Internalising symptoms refer to symptomatic emotional states arising from internalising reactions that involve anxiety and depression symptoms (Lau et al. 2019; Liu et al. 2011). The cognitive model of internalising symptoms proposes that the information processing system consists of bottom‐up threat evaluation and top‐down attentional control (Mogg and Bradley 2016). The threat evaluation is an automatic process where children evaluate how much a stimulus is threatening. Attentional control refers to the voluntary process to effortfully attend to positive or negative information. According to the cognitive models, the tendency to evaluate stimuli as more threatening and to shift attention away from positive stimuli contributes to the development of strong negative attentional bias and weak positive attentional bias, which facilitates the development and maintenance of internalising symptoms (Mogg and Bradley 2016).

According to the transactional model (Field and Lester 2010), child development is defined as the consequence of the interaction between the maturational process intrinsic to children (e.g., the innate development of attentional functions) and the learning process that is susceptible to the influence of factors in the environment (e.g., parenting styles, education). Field and Lester (2010) posited three possible developmental models of negative attentional bias in children and adolescents. The integral model of biased attention posits that different children were born with different levels of attentional bias; depending on personal traits, some children showed high negative attentional bias, and others showed low negative attentional bias. Age does not affect attentional bias, such that the level of biased attention would remain the same throughout a lifetime.

The moderation model of biased attention posits that all children were born with similar levels of attentional bias. Age moderates the development of attentional bias in children, such that their attentional bias might increase or decrease across developmental stages. The acquisition model suggests that children do not have biased attention at birth. Attentional bias was acquired as a consequence of child development. For example, some children might start to develop negative attentional bias when they grow older.

However, compared to other information processing biases (e.g., memory bias and interpretation bias), few published studies have investigated the developmental trajectories of attentional biases in children and adolescents, making it difficult to reach a conclusion on which developmental model of attentional bias is the most accurate.

### 1.2 Attentional Functions Underlying Attentional Biases

Attentional functions underlying attentional biases include sustained attention, inhibitory control, and selective attention (Mogg and Bradley 2016). Thus, based on the improved development of attentional functions during the transition into early adolescence (Betts et al. 2006; Bunge et al. 2002; Kar et al. 2011), healthy children are more likely to display improved attentional functions. With better attentional functions, maladaptive negative attentional bias might be reduced because of a better capability to control attention to positive stimuli during the transition into early adolescence (Liang et al. 2017). However, abnormal attentional functions might increase the risk of abnormal patterns of low or decreasing positive attentional bias and high or increasing negative attentional bias, which may lead to maladaptive developmental trajectories of high or increasing internalising symptoms.

### 1.3 The Developmental Trajectories of Internalising Symptoms During the Transition Into Adolescence

Based on the cognitive theory of internalising symptoms, attentional bias is the vulnerability factor for the development of anxiety and depression symptoms (Mogg and Bradley 2016). The development of internalising symptoms (e.g., anxiety and depression symptoms) in the present study is defined by the trajectories of anxiety and depression symptoms across critical developmental stages that might be affected by the changing patterns of attentional biases.

Research has revealed heterogeneous developmental trajectories of internalising symptoms during the transition into adolescence. These can be categorised into maladaptive and adaptive developmental trajectories. The adaptive developmental trajectories are defined by the overall decreasing or constantly low level of internalising symptoms. While the maladaptive developmental trajectories follow the opposite developmental patterns, including the constantly high or increasing level of internalising symptoms. For example, a study on the developmental trajectories of internalising symptoms during the transition into early adolescence from 9 to 15 years old found two different developmental trajectories for anxiety, including steadily low (82%) and high declining groups (12.1%); the study identified two different developmental trajectories for depression: steadily low (94.1%) and moderate increasing groups (5.9%) (Prinzie et al. 2014). Gender differences were also reported in the trajectories of internalising symptoms in early adolescents. Four trajectories of depressive symptoms were found in girls, but three trajectories were revealed in boys (Fernandez Castelao and Kröner‐Herwig 2013). Thus, based on the previous studies, the majority of children showed adaptive trajectories of low internalising symptoms, while the minority of children had maladaptive trajectories of high or increasing internalising symptoms from childhood into early or middle adolescence. As children undergo significant cognitive development during this critical developmental period, attentional biases may also change over time. Based on the cognitive theory of internalising symptoms, different changing courses of attentional biases may co‐develop with and predict different trajectories of internalising symptoms in children.

However, no studies have investigated the trajectories of attentional biases and the relationship of the developmental patterns of attentional biases to the developmental trajectories of internalising symptoms. This project aims to fill this research gap by establishing developmental trajectories of attentional biases and assessing their association with the developmental trajectories of internalising symptoms during the transition into adolescence. Such findings would contribute to the developmental model of attentional biases and internalising symptoms. The developmental model of attentional bias and internalising symptoms would provide a theoretical framework to support the development of interventions or preventions that help change the maladaptive developmental patterns of internalising symptoms by adjusting the patterns of children and adolescents' attentional biases during the critical period of transition into early adolescence when puberty‐related physical and cognitive changes would bring additional challenges to adolescence.

### 1.4 Active‐Avoidant Coping Styles

Based on Ayers et al. (1996), avoidant coping style refers to avoiding solving problems, and instead adopting methods, including avoidant action, repression, and wishful thinking to escape from them. Active coping style, on the other hand, describes more problem‐focused methods to directly cope with stressors, including cognitive decision‐making, direct problem‐solving, and positive thinking (Ayers et al. 1996). Active and avoidant coping styles are important correlates of internalising symptoms. A study showed that adolescents from Grade 6 reported an increase in depression symptoms if they tended to use an avoidant coping style, while those who tended to use an active coping style showed a decrease in depression symptoms (Herman‐Stahl et al. 1995). A study recruited 168 Latino youth, and the results showed that active coping was related to fewer internalising symptoms and less posttraumatic stress, while avoidant coping was associated with more internalising symptoms and posttraumatic stress (Gudiño et al. 2018).

The extant literature on the relationship between avoidant coping style and negative attentional bias suggests that having an avoidant coping style strengthens attention toward threats at an early stage in adults (Derakshan et al. 2007; Luecken et al. 2004). This suggests that a higher avoidant coping style is related to higher negative attentional bias. Additionally, a higher active coping style is associated with weaker negative attentional bias (Avero et al. 2003). However, the association between avoidant‐active coping style and positive attentional bias was less investigated.

The above literature review showed that active‐avoidant coping styles were associated with both internalising symptoms and negative attentional bias. Thus, active and avoidant coping styles might account for or confound the significant association between internalising symptoms and negative attentional bias; however, this has not been investigated.

The aforementioned literature has demonstrated the importance of attentional biases in the development of internalising symptoms in children. For theoretical reasons, depression and anxiety symptoms are highly comorbid with each other, which can represent the general level of internalising symptoms (Wiggins et al. 2023). The presence of attentional bias is likely to predict both anxiety and depression symptoms (Ho et al. 2018, 2021), which together are better defined as children's internalising symptoms than anxiety symptoms or depression symptoms separately. However, although there are some theories and empirical research to explain the relationship of attentional bias to anxiety and depression, including the work by Field and Lester (2010), not enough studies have been conducted on the general level of internalising symptoms. The inclusion of anxiety and depression symptoms as a unique dimension can reflect the development of internalising symptoms. Thus, the results from such a study can help expand the cognitive theories and the developmental theory of Field and Lester by applying these to explain the development of internalising symptoms. The theoretical contribution of this study is to pave a foundation for a developmental model of attentional bias and internalising symptoms.

Previous studies have shown that gender is a significant predictor of internalising symptoms (Leibenluft 1999). Moreover, women showed stronger attention toward positive cues than men (Kinney et al. 2017). Given the above literature, the effect of gender should be considered in the data analyses.

### 1.5 The Current Research

Altogether, based on the literature review, no studies have used a developmental model to understand (1) what the developmental patterns of negative and positive attentional biases are, (2) how internalising symptoms codevelop with negative and positive attentional biases, and (3) whether active and avoidant coping styles might confound the association between attentional biases and symptoms. The developmental model would help explain how children's development of cognition, affective, and behavioural functions might interact during the critical period of the transition into early adolescence.

From the clinical perspective, the model would imply how we can facilitate healthy development of emotional health during the transition into early adolescence by intervening in children's maladaptive coping styles and attentional patterns. Thus, the model has implications for the prevention and interventions against internalising symptoms for children transitioning into early adolescence.

Based on research gaps identified in the literature review, the current research adopts a longitudinal design to (1) identify heterogeneous developmental trajectories of positive attentional bias and negative attentional bias, (2) test the association of the developmental trajectories of negative and positive attentional biases with the trajectories of internalising symptoms during transition into adolescence, and (3) investigate whether the development of attentional biases significantly predicted the developmental patterns of internalising symptoms beyond the effect of active and avoidant coping styles during transition into early adolescence.

Based on the development of attentional functions in children, during the transition into adolescence, healthy developmental patterns of attentional biases were hypothesized to be characterised by an increasing positive attentional bias and a decreasing negative attentional bias, after controlling for covariates (e.g., gender). The abnormal developmental patterns would show opposite patterns, after controlling for covariates (e.g., gender). Also, it was hypothesized that the development of internalising symptoms would be predicted by the developmental patterns of attentional biases. Moreover, after controlling for the effect of gender, it was hypothesized that the association would become insignificant when considering the effect of active‐avoidant coping styles. Because of the lack of literature on the relationship between active‐avoidant coping styles and positive attentional bias, the study would also explore whether the association between the trajectories of internalising symptoms and the trajectories of positive attentional bias would remain significant after considering the effect of active‐avoidant coping styles.

## 2 Method

### 2.1 Participants

**FIGURE 1:**
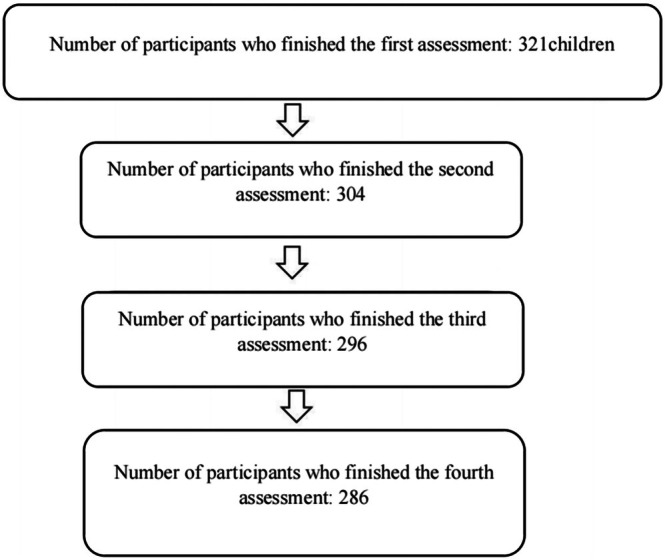
The flowchart of exclusion of participants.

The inclusion criteria were (1) the ability to fluently speak and understand simplified Chinese, and (2) no diagnosis of psychological disorders including anxiety disorders and depression disorders. 12 children did not fulfil the criteria. 24 children did not agree to participate or did not fulfil the criteria. Finally, 326 children participated in the study (N_girls_ = 172, N_boys_ = 147, 7 children did not indicate their gender). Five children did not attend the assessment session at T1 (1.5%). The final sample at T1 consisted of 321 children. Among these children, 172 were girls (52.8%), 147 were boys (45.1%), and 2 children did not indicate their gender. Their age range was from 9 to 10 years (*M* = 9.56, *SD* = −0.50). The majority were born in mainland China (90.2%), and only 28 were born in Hong Kong (8.6%). Seventeen children (5.3%) did not attend the second assessment, because they applied for study leave on that date. In the third assessment, 8 of 304 children (2.6%) who completed the second assessment did not finish the self‐reported scales due to absence from the school on the date of assessment. 10 of 296 children who completed the third assessment (3.4%) did not complete the fourth assessment due to absence from school. In summary, 286 children attended all four assessments. Refer to Figure 1 for the procedure of exclusion of participants.

After data cleaning, 22 children who missed one of the assessment sessions were also excluded. The final dataset comprised 264 students (138 girls, 124 boys, and 2 children who did not indicate gender). Data from these two children who did not report gender were excluded from the analyses, wherever gender was controlled for in the analyses. There is no significant difference between participants who dropped out and those who completed all the assessments. Refer to Section A3 in the appendices.

### 2.2 Procedure

All research procedures were performed under relevant guidelines and regulations of the Human Subjects Ethics Sub‐Committee. The current research conducted assessments in a sample of Grade 4 children four times at a 6‐month interval and over approximately 18 months.

Only students who signed the assent form and obtained a signed copy of the informed consent form from their parents or legal guardians participated in the study. The parents' informed consent and children's assent forms were provided only once at the beginning of the study. In the first assessment session, participants completed a set of questionnaires that included the Attention to Positive and Negative Information Scale (APNIS), Revised Child Anxiety and Depression Scale (RCADS), and translated Children's Coping Strategies Checklist—Revised1 (CCSC‐R1) in classrooms. In the second, third, and fourth assessment sessions, participants completed self‐report inventories to measure their attentional biases and internalising symptoms using the APNIS and RCADS. In each assessment, students were given approximately 40 min to complete all the questionnaires. A researcher and a teacher were present during all assessment sessions to answer the participants' questions and to ensure the safety of students.

### 2.3 Measurement

#### 2.3.1 Active and Avoidant Coping Styles

The active coping subscale and the avoidant coping subscale of the Children's Coping Strategies Checklist‐Revised1 (CCSC‐R1) were used to measure active and avoidant coping styles (Program for Prevention Research 1999). Because the scale does not have a Chinese version, the CCSC‐R1 scale was translated into Chinese (see the translation process in Section A5 of the Appendices). Items (e.g., ‘When you had a problem in the past month, you tried to ignore it’) were rated on a four‐point Likert scale (1 = never, 4 most of the time). The Chinese translated versions of the scales showed satisfactory to excellent internal consistency at T1 (avoidant coping: Cronbach's *α* = 0.74; active coping: Cronbach's *α* = 0.94). Avoidant coping scores and active coping scores were calculated by averaging scores for each item on the avoidant coping scale and the active coping scale. Refer to Section A4 in the Appendices for the Chinese translation of the scale.

#### 2.3.2 Internalising Symptoms

Internalising symptoms were assessed by the Chinese version of the Revised Child Anxiety and Depression Scale (Piqueras et al. 2017). The Anxiety Disorders scale (37 items) has items such as ‘afraid of looking foolish in front of people’, and the Major Depressive Disorder scale (10 items) includes items such as ‘feels nothing is much fun anymore’. Items were rated on a Likert scale from 0 to 3. The scale showed adequate structural validity and internal consistency in Chinese youth (Lu et al. 2021). It had excellent internal consistency for the Anxiety Disorder Scale and good internal consistency for Major Depression Disorder at T1 (Anxiety Disorder scale: Cronbach's *α* = 0.96; Major Depression Disorder scale: Cronbach's *α* = 0.88; Total scale: Cronbach's *α* = 0.96). Similar to previous studies (e.g., Klim‐Conforti et al. 2021), the total internalising symptoms scores were calculated by summing the scores of all items. Adding anxiety and depression symptoms together to represent a unique dimension is methodologically sound for measuring internalising symptoms (Akçay et al. 2022). Greater internalising symptoms were indicated by higher total anxiety and depression scores.

#### 2.3.3 Attentional Biases

APNIS includes items that measure children's tendency to attend to positive or negative information in daily life (Chan et al. 2011; Noguchi et al. 2006). An eight‐item version of APNIS was derived by exploratory factor analysis (EFA) and confirmatory factor analysis (CFA). Please refer to Section A6 in the Appendices for EFA and CFA. The brief scale has satisfactory to acceptable internal consistency in the four assessments of the current study (ANI subscale: Cronbach's *α* = 0.73–0.78; API subscale: Cronbach's *α* = 0.62–0.71). Participants rated each of the eight items on a five‐point Likert scale from one (strongly disagree) to five (strongly agree). ANI scores and API scores were calculated by summing scores from each item in the ANI subscale or API subscale, respectively. The higher the score, the stronger the attentional biases. Refer to A4 for the Chinese translation of the scale.

### 2.4 Statistical Analyses

#### 2.4.1 Preliminary Analyses

Means and standard deviations of psychological variables are presented by gender. The association between psychological variables and age was assessed by Pearson's correlation. Using SPSS version 22, the associations between psychological variables at each assessment session (T1, T2, T3, T4), controlling for gender, were examined by Pearson's partial correlation. A preliminary assessment of longitudinal measurement invariance was conducted to test the comparability of latent constructs over time (refer to Section A8 in Appendices).

#### 2.4.2 Trajectories of Negative and Positive Attentional Biases

Latent class growth analysis (LCGA) was performed in Mplus 7.11 (Muthén and Muthén 2012) to identify trajectories of attentional biases. A series of models were tested, beginning with the 1‐class model (refer to Sections A1 and A2 in the appendices).

We tested conditional models with covariates and unconditional models without covariates separately. Based on the previous literature, the conditional model which includes significant covariates for the trajectories tended to perform better; the unconditional model without the covariates is likely to lead to distorted results (Jung and Wickrama 2008). Also, refer to Sections A1 and A2 in the appendices which showed that the conditional model outperformed the unconditional model. Thus, conditional models of LCGA were conducted twice to identify distinct trajectories of negative attentional bias and positive attentional bias, considering the effects of gender, active and avoidant coping, anxiety symptoms, and depression symptoms at baseline (T1). Refer to Section A9 in the appendices for gender differences in the trajectories.

It should be noted that the conditional model of trajectories and the joint trajectory analyses where the trajectory is conditional on another trajectory are two separate analytical methods to answer different research questions. The conditional models added covariates to increase the model fit of trajectories. This step is to answer the research question on the identification of the trajectories of attentional biases. However, this method cannot answer the research questions related to how the developmental patterns of positive or negative attentional biases are associated with the development of internalising symptoms. To answer this question, the joint trajectory analyses where we tested whether and how the trajectories of attentional biases were conditioned on the trajectories of internalising symptoms were subsequently conducted.

#### 2.4.3 Joint Trajectories of Attentional Biases and Internalising Symptoms

Because the conditional model had better fit indices (refer to Sections A1 and A2), the same LCGA procedure (conditional model) was conducted to reveal the developmental trajectories of internalising symptoms, controlling for covariates including negative and positive attentional biases, gender, and active and avoidant coping styles at baseline (T1). It should be noted that our primary focus was on characterising the developmental patterns of attentional biases within a theoretically grounded framework. The inclusion of these covariates is important to establish an accurate developmental model of attentional biases and internalising symptoms, taking into account the potential confounding effect of the covariates, which paves a better foundation for the subsequent joint trajectory analyses.

Then, joint trajectory analyses were performed according to the previously reported procedure (Haltigan and Vaillancourt 2014; Vaillancourt et al. 2017) to assess the association between the trajectories of internalising symptoms and the trajectories of attentional biases. Two sets of key probabilities for joint trajectories were reported: joint probabilities and conditional probabilities (Brame et al. 2001). The joint probabilities indicate the probabilities of showing the joint trajectory group of attentional biases/internalising symptoms. The conditional probabilities indicate the probabilities of children belonging to a trajectory group for internalising symptoms, conditional on their trajectory group for negative attentional bias or positive attentional bias.

#### 2.4.4 The Predictive Value of Trajectories of Attentional Biases

To understand whether the trajectory membership of negative attentional bias and the trajectory membership of positive attentional bias predict the trajectory membership of internalising symptoms, beyond active and avoidant coping styles, ordinal logistic regression analyses were performed; controlling for the effect of active and avoidant coping styles.

## 3 Results

### 3.1 Preliminary Analyses

The demographic statistics (means and standard deviations) for each psychological variable across the four assessment waves (T1, T2, T3, T4) by gender are presented in Table 1. Independent t‐tests were performed to examine gender differences in the psychological variables. Girls showed significantly higher internalising symptoms at T3, *t* (260) = −2.85, *p* < 0.01, and T4, *t* (260) = −2.83, *p* < 0.01, than boys. Moreover, girls showed significantly higher negative attentional bias at T1, *t* (260) = −2.81, *p* < 0.01, T2, *t* (260) = −2.23, *p* < 0.05, and T4, *t* (260) = −2.69, *p* < 0.01. Refer to Table 2 for the correlation among the variables, controlling for gender. Age was not significantly related to any of the psychological variables, *r*s = −0.00 to 0.10, *ps* = ns.

**TABLE 1:** Mean (standard deviation) of psychological variables by gender.

	Means	SD	Means	SD	Means	SD	*t*
	Total		Girls		Boys		
Time 1							
1. T1 Active Coping	2.55	0.69	2.49	0.65	2.61	0.72	1.41
3. T1 Avoidant Coping	1.59	0.60	1.62	0.56	1.55	0.64	−0.86
5. T1 ANI	11.09	4.06	11.73	3.98	10.34	4.04	−2.81**
6. T1 API	14.5	3.14	14.20	3.39	14.87	2.80	1.73
7. T1 RCADS	31.28	26.02	33.99	27.33	28.00	24.30	−1.87
Time 2							
8. T2 ANI	10.90	4.02	11.41	3.98	10.31	3.99	−2.23*
9. T2 API	14.92	3.24	14.79	3.18	15.06	3.33	0.66
10. T2 RCADS	14.02	18.15	15.28	19.13	12.58	17.05	−1.20
Time 3							
11. T3 ANI	11.16	3.92	11.51	4.05	10.80	3.75	−1.48
12. T3 API	14.87	3.26	14.86	3.09	14.88	3.46	0.04
13. T3 RCADS	31.08	25.92	35.30	28.43	26.27	22.15	−2.85**
Time 4							
14. T4 ANI	11.75	4.12	12.41	4.40	11.06	3.69	−2.69**
15. T4 API	14.81	3.38	14.94	3.31	14.66	3.46	−0.67
16. T4 RCADS	31.50	25.40	35.62	26.81	26.85	23.01	−2.83**

*Note:* *p < 0.05; **p < 0.01.

Abbreviations: ANI, attention to negative information; API, attention to positive information; T1, time one; T2, time two; T3, time three; T4, time four.

**TABLE 2:** Correlation among psychological variables at T1, T2, T3, T4, controlling for gender.

Correlations (rs)																				
	1	2	3	4	5	6	7	8	9	10	11	12	13	14	15	16	17	18	19	20
T1																				
1. ANI	—	−0.06	0.54**	0.47**	0.54**	0.45**	−0.10	0.38**	0.29**	0.37**	0.42**	−0.21**	0.45**	0.40**	0.45**	0.42**	−0.14*	0.41**	0.36**	0.41**
2. API		—	−0.23**	−0.33*	−0.26**	−0.20**	0.33**	−0.18**	−0.19**	−0.18**	−0.16**	0.32**	−0.13*	−0.18**	−0.14*	−0.11	0.30**	−0.13*	−0.12*	−0.13*
3. ANX			—	0.85**	0.99**	0.46**	−0.14*	0.62**	0.50**	0.61**	0.39**	−0.21**	0.59**	0.56**	0.60**	0.40**	−0.14*	0.54**	0.49**	0.55**
4. MDD				—	0.90**	0.44**	−0.21**	0.55**	0.54**	0.56**	0.33**	−0.22**	0.52**	0.58**	0.54**	0.33**	−0.20**	0.47**	0.48**	0.48**
5. RCADS					—	0.46**	−0.15*	0.62**	0.52**	0.62**	0.39**	−0.22**	0.59**	0.58**	0.60**	0.39**	−0.15*	0.54**	0.50**	0.55**
T2																				
6. ANI						—	−0.17**	0.49**	0.41**	0.49**	0.59**	−0.18**	0.49**	0.49**	0.50**	0.51**	−0.18**	0.46**	0.45**	0.47**
7. API							—	−0.14*	−0.18**	−0.15*	−0.20**	0.42**	−0.23**	−0.24**	−0.24**	−0.12	0.43**	−0.24**	−0.23**	−0.24**
8. ANX								—	0.85**	0.99**	0.47**	−0.14*	0.68**	0.64**	0.69**	0.37**	−0.16**	0.55**	0.53**	0.56**
9. MDD									—	0.90**	0.34**	−0.13*	0.53**	0.60**	0.55**	0.28**	−0.20**	0.43**	0.47**	0.45**
10. RCADS										—	0.45**	−0.15*	0.67**	0.65**	0.68**	0.36**	−0.17**	0.54**	0.53**	0.55**
T3																				
11. ANI											—	−0.11	0.68**	0.62**	0.68**	0.55**	−0.15*	0.50**	0.46**	0.51**
12. API												—	−0.18**	−0.22**	−0.19**	−0.17**	0.55**	−0.18**	−0.22**	−0.20**
13. ANX													—	0.86**	0.99**	0.51**	−0.16*	0.72**	0.60**	0.71**
14. MDD														—	0.91**	0.43**	−0.21**	0.65**	0.64**	0.66**
15. RCADS															—	0.51**	−0.17**	0.72**	0.62**	0.72**
T4																				
16. ANI																—	−0.10	0.60**	0.53**	0.60**
17. API																	—	−0.23**	−0.29**	−0.25**
18. ANX																		—	0.85**	0.99**
19. MDD																		.	—	0.91**
20. RCADS																				—

*Note:* *p < 0.05; **p < 0.001.

Abbreviations: ANI, attention to negative information; ANX, anxiety; API, attention to positive information; MDD, major depression disorder; RCADS, anxiety and depression total symptoms; T1, time one; T2, time two; T3, time three; T4, time four.

Measurement invariance analyses indicated that configural, metric, and scalar invariance were mostly supported for the Attention to Positive Information (API) and Attention to Negative Information (ANI) scales as well as the Revised Child Anxiety and Depression Scale (RCADS) (see Section A8 for details).

### 3.2 Developmental Trajectories of Attentional Biases

The model fit indices showed that a model of multiple trajectories would be better to represent the changes in negative attentional bias, positive attentional bias, and internalising symptoms (refer to Section A1 in appendices). Also, LCGA with measurement models has poorer model fit indices (refer to Section A7 in appendices). Thus, using measurement models in LCGA would produce less accurate results of trajectory groups, which would negatively impact the findings of subsequent analyses. Therefore, results from LCGA with total scores of attentional biases and internalising symptoms were adopted and presented.

#### 3.2.1 Negative Attentional Bias Trajectories

Including gender, active and avoidant coping styles, anxiety symptoms, and depression symptoms at T1 in the model, LCGA revealed that the nonchanged 3‐class model showed the best model fit (refer to Table A1.1 in Appendices).

**FIGURE 2:**
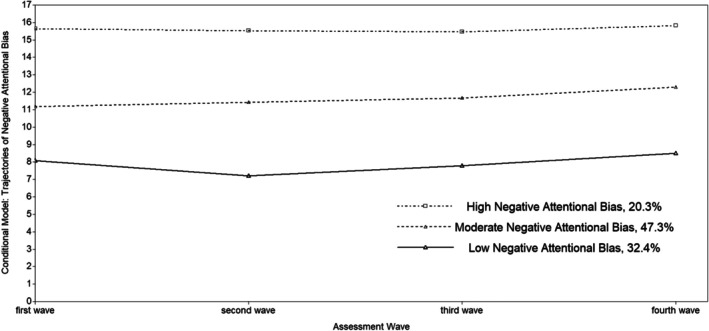
The developmental trajectories of negative attentional bias (conditional model).

The data are shown in Figure 2. Class one was the ‘low negative attentional bias’ trajectory group (32.4%), which was characterised by the lowest level of negative attentional bias throughout the study period (mean intercept = 7.82, *p* < 0.001). Group two was defined as the ‘moderate negative attentional bias’ trajectory group, which consisted of 47.3% of the participants. The trajectory group had a moderate level of negative attentional bias throughout the study period (mean intercept = 11.61, *p* < 0.001). Group three was the ‘high negative attentional bias’ trajectory group (20.3%), which had the highest level of negative attentional bias throughout the study period (mean intercept = 15.59, *p* < 0.001). The three trajectories of negative attentional bias showed a stable nonchanged pattern throughout the transition from childhood into early adolescence.

#### 3.2.2 Positive Attentional Bias Trajectories

**FIGURE 3:**
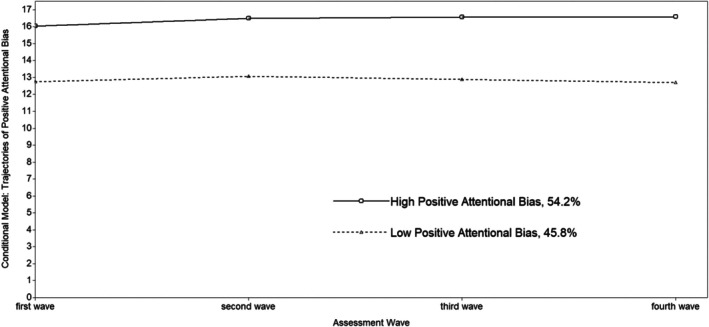
The developmental trajectories of positive attentional bias (conditional model).

LCGA for positive attentional bias, controlling for the effect of gender, active and avoidant coping, anxiety, and depression symptoms, showed that the 2‐class model had the best model fit among the 1‐class to the 4‐class (refer to Table A1.2 in appendices). The 2‐class nonchanged model was selected. According to Figure 3, class one had consistently high positive attentional bias, and was defined as the ‘high positive attentional bias’ group which had 54.2% of the participants (mean intercept = 16.41, *p* < 0.001). Class two was the ‘low positive attentional bias’ group (45.8%) which was characterised by a consistently low positive attentional bias from T1 to T4 (mean intercept = 12.85, *p* < 0.001). The two trajectories of positive attentional bias showed a stable nonchanged pattern throughout the transition from childhood into early adolescence.

### 3.3 The Association Between Attentional Biases Trajectories and Internalising Symptoms Trajectories

#### 3.3.1 Internalising Symptoms Trajectories

The trajectories of internalising symptoms (total anxiety and depression scores) in early adolescents were examined by LCGA (conditional model), controlling for the effects of gender, negative and positive attentional biases, and active and avoidant coping styles at baseline (T1). The 3‐class nonlinear model was selected because it had the best mode fit (refer to Table A1.3 in appendices).

**FIGURE 4:**
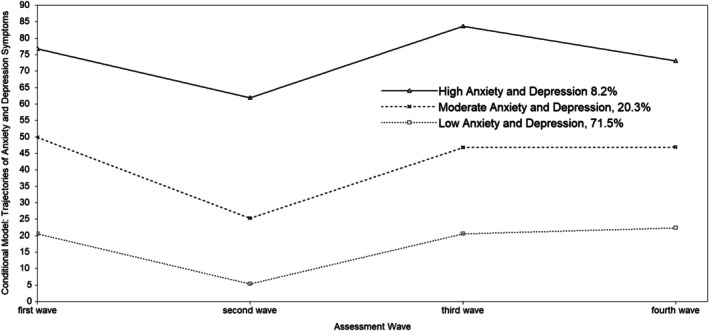
The developmental trajectories of internalising symptoms (conditional model).

Figure 4 shows the trajectories of internalising symptoms. Class one was the ‘low symptoms’ trajectory group (71.5%), which was characterised by a low level of internalising symptoms throughout the study period (mean _intercept_ = 17.80, *p* < 0.001, mean _quadratic slope_ = 6.79, *p* < 0.001). Class two was the ‘moderate symptoms’ trajectory group (20.3%), which showed a moderate level of internalising symptoms throughout the study period (mean _intercept_ = 45.65, *p* < 0.001, mean _quadratic slope_ = 10.06, *p* < 0.001). Class three was the ‘high symptoms’ trajectory group (8.2%), which had a high level of internalising symptoms throughout the study period (mean _intercept_ = 72.45, *p* < 0.001, mean _quadratic slope_ = 5.05, *p* = 0.07).

The trajectory of ‘low internalizing symptoms’ started from the lowest level of internalising symptoms. Based on the positive quadratic slope, the trajectory exhibited the shape of a quadratic nonlinear trend where the level first decreased from T1 to T2, and then increased from T2 to T4. The trajectory of ‘moderate internalizing symptoms’ started at the moderate level of internalising symptoms. It had the shape of a quadratic nonlinear trend where the symptoms first decreased from T1 to T2, and then increased from T2 to T4. The trajectory of ‘high internalizing symptoms’ started from the highest level of internalising symptoms; the symptoms first decreased from T1 to T2 and then increased from T2 to T3. Then, it decreased again to the level of the starting point from T3 to T4.

#### 3.3.2 Joint Trajectory Analysis for Negative Attentional Bias

The *χ* ^2^ test indicated that the difference in the joint probabilities was significant, *χ* (4)^2^ = 133.99, *p* < 0.001. There was a 3 × 3 array of joint trajectory groups for negative attentional bias and internalising symptoms. Children were most probable to be in the low negative attentional bias/low symptoms group (36%, *N* = 96). Fewest participants were in the moderate negative attentional bias/high symptoms developmental group (0%, *N* = 0).

For the conditional probabilities, children showing a low negative attentional bias trajectory were most likely to show the low symptoms trajectory group (78.7%). Children who displayed the moderate negative attentional bias trajectory group were most likely to have the low symptoms trajectory (97.7%). Children who showed the trajectory group of high negative attentional bias were most likely to exhibit the high symptom trajectory pattern (86.4%).

#### 3.3.3 Joint Trajectory Analyses for Positive Attentional Bias

The *χ* ^2^ test suggested that the difference in the joint probabilities was significant, *χ* (2)^2^ = 22.621, *p* < 0.001. With the trajectory memberships of positive attentional bias and internalising symptoms as starting points, 2 × 3 joint trajectory groups for positive attentional bias and symptoms were presented. Children were most probable to be in the high positive attentional bias/low symptoms group (45.8%, *N* = 120). The fewest participants followed the high positive attentional bias/high symptoms developmental path (3.1%, *N* = 8).

For the conditional probabilities, children in the trajectory group of high positive attentional bias were significantly more probable to be in the trajectory group of low symptoms than in the moderate (11.1%) and high symptoms groups (5.6%). Also, children in the trajectory group of high positive attentional bias were more likely to be in the low symptoms group (83.3%) than children in the trajectory group of low positive attentional bias (56.8%).

### 3.4 The Predictive Value of Attentional Bias Trajectories for the Trajectories of Internalising Symptoms, Controlling for Coping Styles

The study found three trajectories of internalising symptoms which represent low, moderate, and high levels of symptoms, respectively, over the 18 months. Thus, a logistic regression analysis was conducted with the three levels of internalising symptoms (low, moderate, high) as the dependent variable, and gender, active and avoidant coping styles, negative and positive attentional biases as independent variables (refer to Table 3). First, the trajectory membership of negative and positive attentional biases and gender were input in the model. Trajectory membership of higher negative attentional bias significantly predicted a higher internalising symptoms trajectory over the period. Also, the trajectory membership of higher positive attentional bias significantly predicted the trajectory membership of lower internalising symptoms. After inputting active and avoidant coping styles into the model, the model remained significant, *χ* ^2^ (5) = 104.726, *p* < 0.001. *Negative* and positive attentional biases remained significant predictors. Thus, the trajectory membership of negative and positive attentional biases could explain the variance of the internalising symptoms trajectory beyond the contributions of active and avoidant coping styles.

**TABLE 3:** Regression analysis for the relationship of attentional biases to internalising symptoms, controlling for coping.

	Estimate	Walf	*p*
Gender	−0.507	2.328	0.127
Avoidant coping	0.966	13.744	< 0.001
Active coping	−0.406	1.990	0.158
ANI (trajectory groups)	1.284	36.780	< 0.001
API (trajectory groups)	−1.176	9.014	0.003

Abbreviations: ANI, attention to negative information; API, attention to positive information.

## 4 Discussion

### 4.1 The Development of Positive Attentional Bias in Children During Transition Into Early Adolescence

One of the aims of the present study is to identify the developmental trajectories of attentional biases. Consistent with the hypothesis, for a large proportion of children, the development of positive attentional bias was healthy as it was constantly high. A minor proportion of children held a low level of positive attentional bias. Although sustained attention, inhibitory control, and selective attention were developing during the transition into early adolescence (e.g., Betts et al. 2006), inconsistent with the hypothesis, the developmental trajectories of positive attentional bias remained stable across the period. Thus, the results might suggest that the self‐report cognitive tendency to attend to positive information might be less likely to be affected by the development of attentional functions. Based on the relationship of self‐reported positive attentional bias to dispositional optimism, positive affectivity, and extraversion (Noguchi et al. 2006), the more stable personality traits related to optimism, positive affectivity, and extraversion may play a significant role in deciding the level of positive attentional bias.

Based on the three developmental models by Field and Lester (2010), the integral model suggests that the level of attentional bias would remain the same across child development. The results of the trajectory analyses for positive attentional bias provided empirical evidence for the integral model of attentional bias proposed by Field and Lester (2010). Although the integral model was originally proposed to explain the development of negative attentional bias, the present findings showed that this integral model can be applied to the development of attention towards positive information during the transition into early adolescence.

### 4.2 The Development of Negative Attentional Bias During the Transition Into Adolescence

One of the study's aims was to investigate the trajectory of negative attentional bias. Inconsistent with the hypotheses, the results suggested that the level of negative attentional bias showed a constant level across the period, as minimal changes in all three trajectory groups were observed. This was inconsistent with a previous study that found other maladaptive negative cognitive biases, including threat interpretation, negative attributions, and overgeneralising, significantly increased in children from 10 to 17 years of age (Slavny et al. 2019). Notably, previous studies recruited adolescents who were older than the participants in the current study. Additionally, the assessment period in the previous study was longer than the 18 months of the current study. These factors may contribute to the inconsistency.

Based on the three developmental models in the previous literature by Field and Lester (2010) mentioned above, the present findings on the trajectory of negative attentional bias provided support to the integral model, which suggested that the level of negative attentional bias maintains at a consistent level across child development. Despite previous literature showing that children's attentional functions undergo significant changes (Betts et al. 2006; Ickx et al. 2017), the findings of the current study suggested that the developmental trajectories of negative attentional bias were relatively stable throughout the 18 months, with very small to no increase. Negative attentional bias refers to the cognitive tendency to focus on negative information and is significantly related to personality traits and temperament, including lower optimism, higher negative affectivity, and higher neuroticism (Noguchi et al. 2006). Thus, the trajectories of self‐reported attentional bias might be affected by children's dispositional factors, such as personality and temperament, which were less likely to be affected by the development of attentional functions.

### 4.3 The Association Between Developmental Trajectories of Attentional Biases and Those of Internalising Symptoms

An important purpose of the study was to understand how trajectories of internalising symptoms codeveloped with the trajectories of attentional biases, which aimed to add a developmental perspective to the cognitive model of internalising symptoms. The cognitive model and previous empirical findings related to internalising symptoms and attentional biases suggested that high negative attentional bias and low positive attentional bias are associated with and are likely to predict the abnormal trajectory of higher internalising symptoms (Abend et al. 2018; Ho et al. 2018; Mogg and Bradley 2016). Consistent with our hypotheses, the present findings suggested that across the critical developmental stages of transition into early adolescence, the abnormal developmental patterns of internalising symptoms could be predicted by the trajectory of constantly low positive and high negative attentional bias. This answered the research question on the association between the development of internalising symptoms and the developmental patterns of attentional biases. These findings not only provide more support to the previous literature on the cognitive model of attentional biases and internalising symptoms but also add a developmental perspective to the model of attentional biases and internalising symptoms.

Previous studies showed that active and avoidant coping styles were associated with both attentional biases and internalising symptoms (Avero et al. 2003; Derakshan et al. 2007; Herman‐Stahl et al. 1995). However, inconsistent with our hypothesis, results from the ordinal logistic regression indicated that the association between attentional biases and internalising symptoms was still significant even if active and avoidant coping styles were accounted for. This suggested that the association between negative and positive attentional bias and internalising symptoms was significant beyond the effect of active and avoidant coping styles.

## 5 Implications

The study built a developmental model of attentional biases and internalising symptoms. Based on this model, children who have shown high negative attentional bias or have shown low positive attentional bias at the beginning are more likely to display the maladaptive trajectory of high internalising symptoms. Moreover, the findings from the present study supported the integral developmental model proposed by Field and Lester (2010). According to the integral model, the level of negative or positive attentional bias is consistent and would not gradually change across the period. Although children's attentional functions are improving, children are less likely to outgrow the maladaptive developmental pattern of high negative attentional bias and low positive attentional bias with child development during the transition into early adolescence. Thus, it might be important to screen high‐risk children who are more likely to have high internalising symptoms based on their level of self‐reported negative and positive attentional bias as soon as possible at the beginning of the transition. Also, findings highlight the importance of active prevention strategies that reduce high self‐report negative attentional bias and increase low self‐report positive attentional bias to help mitigate the risk of high internalising symptoms in children transitioning into early adolescence.

## 6 Limitations

One of the limitations of the study lies in its use of a self‐reported inventory as the only measurement to assess attentional biases, which is subjective to biases. Thus, the results might not be able to generalise to attentional biases measured by behavioural tasks. It might be important to understand whether the long‐term developmental trajectories of attentional biases measured by behavioural tasks or eye‐tracking techniques would be different from the trajectories of self‐reported attentional biases. Another limitation is that the scalar longitudinal invariance for the measure of internalising symptoms was partially supported (see Section A8 in the appendices), which may limit the longitudinal comparability of internalising symptoms across time; thus, its longitudinal comparison should be interpreted with caution. Moreover, the study recruited children from 9 to 11 years of age. It is unknown how children's attentional biases develop over a longer time. Due to the unique developmental stage of transition into early adolescence, the results might not generalise to children of other age ranges or at other developmental periods. Additionally, we only recruited Chinese children. It is unknown whether cultural factors might moderate the results.

## Ethics Statement

The study was approved by the Human Subjects Ethics Sub‐Committee of the City University of Hong Kong (2020‐2021‐CIR2‐B1). The study was performed in accordance with the ethical standards as laid down in the 1964 Declaration of Helsinki and its later amendments or comparable ethical standards.

## Consent

Students' parents who agreed to their children's participation had signed the informed consent sheets, and students had signed the assent sheets to participate.

## Conflicts of Interest

The authors declare no conflicts of interest.

## Supporting information


**Appendix S1:** Supporting Information.

## Data Availability

The datasets generated during and/or analysed during the current study are available after seeking permission from the corresponding author.
