# Supplementary material for: The Developmental Trajectories of Attentional Biases and Their Association With Internalising Symptoms in Children Transitioning Into Early Adolescence
Source: Int J Psychol. 2025 Aug 23;60(5):e70104. doi: 10.1002/ijop.70104 (PMC12374246; doi:10.1002/ijop.70104)
Supplement: Supplementary file 1 — Appendix S1: Supporting Information. [file IJOP-60-e70104-s001.docx]

**Appendices**

**A1. Model Selection of Negative Attentional Bias, Positive Attentional Bias, and Internalizing Symptoms (Conditional Models)**

**Model Selection Criteria**

The best fitting model was chosen based on the following indices (refer to A1-A2 in the appendices): a) Bayesian Information Criterion (BIC), b) Akaike Information Criteria (AIC), c) the Bootstrapped Likelihood Ratio Test (BLRT), d) the Vuong–Lo–Mendell–Rubin Likelihood Ratio (VLMR) Test , e) the Adjusted Lo–Mendell–Rubin Likelihood Ratio (Adj. LMR) Test, f) entropy, and g) having a sufficient number of members in each group (Nguena Nguefack, Pagé, Choinière, & Lacasse, 2019).

Lower BIC and AIC values indicated a better model fit. Significant p values in the BLRT, VLMR, and Adj. LMR tests (p<0.05) indicated that the trajectory model with k class is a better fit to the data compared to the k – 1 class model (Nguena Nguefack, Pagé, Choinière, & Lacasse, 2019). Finally, the model has a good fit if the value of entropy (range 0–1) is closer to 1 (Nguena Nguefack et al., 2019). An increasing number of classes was tested until Latent Class Growth Analysis (LCGA) showed that the k class model was not better than the k-1 class model.

The choice of the model of trajectories is based on the overall consideration of different model fit indices. Among these model fit indices, BIC, a significant Lo, Mendell, and Rubin (2001) likelihood ratio test (LMR) statistic, and the bootstrap likelihood ratio test (BLRT) were suggested to perform better than other model fit indices (Jung & Wickrama, 2008).

**Table A1.1**

*Fit Statistics for Latent Class Growth Analysis Models Representing One to Four Trajectory Groups of Negative Attentional Bias (Conditional Model)*

| Group number  *Shape* | BIC | AIC | VLMR  (p Value) | Adj. LMR  (p Value) | BLRT  (p Value) | Entropy |
| --- | --- | --- | --- | --- | --- | --- |
| 1  *No change* | 10391.27 | 10351.94 | NA | NA | NA | NA |
| 1  *Linear* | 10392.95 | 10350.04 | NA | NA | NA | NA |
| 1  *Nonlinear* | 10396.05 | 10349.56 | NA | NA | NA | NA |
| 2  *No change* | 5620.52 | 5584.83 | .00 | .00 | .00 | 0.81 |
| 2  *Linear* | 5623.36 | 5580.54 | .00 | .00 | .00 | 0.81 |
| 2  *Nonlinear* | 5629.99 | 5580.03 | .00 | .00 | .00 | 0.81 |
| 3  *No change* | **5590.22** | **5522.42** | **.00** | **.01** | **.00** | **0.76** |
| 3  *Linear* | 5596.21 | 5517.70 | .01 | .01 | .00 | 0.77 |
| 3  *Nonlinear* | 5602.86 | 5513.65 | .01 | .01 | .00 | 0.78 |
| 4  *No change* | 5595.97 | 5524.60 | .01 | .01 | .00 | 0.80 |
| 4  *Linear* | 5613.16 | 5527.52 | .56 | .56 | .00 | 0.78 |
| 4 *Nonlinear* | 5621.24 | 5521.33 | .06 | .07 | .33 | 0.82 |

*Note.* The bolded three-group model showed the best model fit.

***Justification of the Choices of the Model for Negative Attentional Bias***

Compared to other models, the 3-class model had the lowest BIC and AIC values among all the classes. The 3-class model also had significant *p* values for the VLMR test, Adj. LMR test and bootstrap likelihood ratio test (BLRT). Thus, the 3-class model was selected. Moreover, among the non-change model, the linear change model, and the nonlinear change model, the non-change model had the lowest BIC. BIC and BLRT are model fit indices that perform better than other indices (Jung & Wickrama, 2008), and thus these two indices were given higher weight when making the decision. Also, based on the graph, the changes in the negative attentional bias also appears to be minimal. Thus, the 3-class non-change model was selected as the best model.

**Table A1.2**

*Fit statistics for Latent Class Growth Analysis Models Representing One to Four Trajectory Groups of Positive Attentional Bias (Conditional)*

| Number of groups  *Shape* | BIC | AIC | VLMR  (p Value) | Adj.LMR  (p Value) | BLRT  (p Value) | Entropy |
| --- | --- | --- | --- | --- | --- | --- |
| 1  *No change* | 10981.69 | 10928.05 | NA | NA | NA | NA |
| 1  *Linear* | 10986.50 | 10929.29 | NA | NA | NA | NA |
| 1  *Nonlinear* | 10990.87 | 10930.08 | NA | NA | NA | NA |
| 2  *No change* | **5289.68** | **5246.86** | **.00** | **.00** | **.00** | **.71** |
| 2  *Linear* | 5296.56 | 5246.60 | .00 | .00 | 0.00 | .72 |
| 2  *Nonlinear* | 5305.85 | 5248.76 | .00 | .00 | .00 | .72 |
| 3  *No change* | 5273.53 | 5205.73 | .08 | .09 | .00 | .69 |
| 3  *Linear* | 5308.22 | 5229.71 | .01 | .00 | .00 | .86 |
| 3  Nonlinear | 5721.90 | 5632.69 | .00 | .00 | .00 | 1.00 |
| 4  *No change* | 5302.05 | 5209.28 | .14 | .14 | NA | .76 |
| 4  *Linear* | 5335.56 | 5228.51 | .54 | .54 | .38 | .82 |
| 4  *Nonlinear* | 5767.29 | 5645.97 | .00 | .00 | .00 | .84 |

*Note.* The bolded two-group model showed the best model fit.

**Justification of the Choices of the Model for Positive Attentional Bias**

Although the 3-class non-change model had the lowest BIC and AIC, it had non-significant p vales for the VLMR test, and the Adj. LMR test, suggesting that the 3-class model was not significantly better than the 2-class model. Also, the 3-class non-change model had the lowest entropy. Thus, it was not selected. Compared to other models, the 2-class nonchanged model had the second lowest BIC values among all the classes. The 2-class model also had significant *p* values for the VLMR test, Adj. LMR test and bootstrap likelihood ratio test (BLRT), suggesting that it was significantly better the general 1-class model. BIC, LMR and BLRT are indices that perform better than other indices. Thus, the 2-class non-change model was selected as the best model.

**Table A1.**

*Fit Statistics for Latent Class Growth Analysis Models Representing One to Four Trajectory Groups of Internalizing Symptoms (Conditional model)*

| Number of groups *Shape* | BIC | AIC | VLMR  (p Value) | Adj. LMR  (p Value) | BLRT  (p Value) | Entropy |
| --- | --- | --- | --- | --- | --- | --- |
| 1  *No change* | 14127.29 | 14073.65 | NA | NA | NA | NA |
| 1  *Nonlinear* | 14120.11 | 14062.90 | NA | NA | NA | NA |
| 1  *Linear* | 14073.19 | 14012.40 | NA | NA | NA | NA |
| 2  *No change* | 9358.49 | 9315.67 | 0.00 | 0.00 | 0.00 | 0.95 |
| 2  *Nonlinear* | 9269.76 | 9212.67 | 0.00 | 0.00 | 0.00 | 0.96 |
| 2  *Linear* | 9350.82 | 9300.86 | 0.00 | 0.00 | 0.00 | 0.96 |
| 3  *No change* | 9315.50 | 9247.70 | 0.11 | 0.12 | 0.00 | 0.80 |
| 3  *Nonlinear* | **9192.70** | **9103.49** | **0.01** | **0.01** | **0.00** | **0.93** |
| 3  *Linear* | 9322.83 | 9244.33 | 0.01 | 0.00 | 0.00 | 0.97 |
| 4  *No change* | 9354.48 | 9261.70 | 0.50 | 0.50 | 1.00 | 0.84 |
| 4  *Nonlinear* | 9233.13 | 9111.80 | 0.01 | 0.01 | 0.11 | 0.95 |
| 4  *Linear* | 9360.53 | 9253.48 | 0.50 | 0.50 | 1.00 | 0.83 |

*Note.* The bolded three-group model showed the best model fit.

**Justification of the Choices of the Model for Internalizing Symptoms**

Compared to all other models, the 3-class nonlinear model had the lowest AIC and BIC values. Additionally, it had a relatively higher entropy. The p-values for VLMR, Adj. LMR and BLRT were all significant. Thus, the 3-class nonlinear model was selected.

**A2. The Model Indices for the Trajectories of Negative Attentional Bias, Positive Attentional Bias, and Internalizing Symptoms (Unconditional Models)**

The unconditional version was conducted without controlling for gender, anxiety, depression, or coping styles, to compare whether the unconditional model was better or worse than the conditional version in terms of their model fit indices. The same selection criteria (e.g., lower BIC indicates better model fit) applied to the unconditional models. As shown in the tables below, the conditional models were superior to the unconditional models.

**Table A2.1**

*Fit Statistics for Latent Class Growth Analysis Models Representing One to Four Trajectory Groups of Negative Attentional Bias (Unconditional)*

| Group number  *Shape* | BIC | AIC | VLMR  (p Value) | Adj. LMR  (p Value) | BLRT  (p Value) | Entropy |
| --- | --- | --- | --- | --- | --- | --- |
| 1  *No change* | 5970.77 | 5952.88 | NA | NA | NA | NA |
| 1  *Linear* | 5972.45 | 5950.99 | NA | NA | NA | NA |
| 1  *Nonlinear* | 5975.55 | 5950.52 | NA | NA | NA | NA |
| 2  *No change* | 5726.37 | 5701.34 | .00 | .00 | .00 | 0.76 |
| 2  *Linear* | 5729.54 | 5697.36 | .00 | .00 | .00 | 0.77 |
| 2  *Nonlinear* | 5736.56 | 5697.22 | .00 | .00 | .00 | 0.77 |
| 3  *No change* | **5691.05** | **5658.87** | **.06** | **.07** | **.00** | **0.71** |
| 3  *Linear* | 5697.31 | 5654.40 | .05 | .06 | .00 | 0.70 |
| 3  *Nonlinear* | 5703.12 | 5649.48 | .11 | .11 | .00 | 0.73 |
| 4  *No change* | 5687.77 | 5648.43 | .00 | .00 | .00 | 0.68 |
| 4  *Linear* | 5695.54 | 5641.90 | .01 | .01 | .00 | 0.71 |
| 4  *Nonlinear* | 5707.03 | 5639.09 | .09 | .10 | .00 | 0.68 |

*Note.* The bolded three-group model showed the best model fit.

**Justification of the Model Choice for Negative Attentional Bias**

Model selection involved comparing fit indices and statistical tests across different numbers of classes and trajectory shapes. First, it demonstrated the lowest BIC value among 3-class models (5691.05), indicating optimal balance between model fit and complexity. Second, the bootstrap likelihood ratio test (BLRT) strongly supported the 3-class solution over simpler alternatives (p < .001), while the VLMR and Adj. LMR tests, though marginally significant (p = .06 and .07), suggested adequate model distinction. Third, the model showed good classification quality with an entropy of 0.71. Most importantly, the stable, no-change trajectory pattern is theoretically consistent with Field and Lester’s (2010) integral model of attentional bias development, which posits that individual differences in attentional bias remain relatively constant across developmental periods rather than showing systematic change with age.

**Table A2.2**

*Fit Statistics for Latent Class Growth Analysis Models Representing One to Four Trajectory Groups of Positive Attentional Bias (Unconditional)*

| Group number  *Shape* | BIC | AIC | VLMR  (p Value) | Adj. LMR  (p Value) | BLRT  (p Value) | Entropy |
| --- | --- | --- | --- | --- | --- | --- |
| 1  *No change* | 5514.53 | 5496.65 | NA | NA | NA | NA |
| 1  *Linear* | 5519.34 | 5497.89 | NA | NA | NA | NA |
| 1  *Nonlinear* | 5523.71 | 5498.68 | NA | NA | NA | NA |
| 2  *No change* | 5374.76 | 5349.73 | .00 | .00 | .00 | 0.64 |
| 2  *Linear* | **5373.66** | **5341.48** | **.00** | **.00** | **.00** | **0.68** |
| 2  *Nonlinear* | 5383.19 | 5343.85 | .00 | .00 | .00 | 0.68 |
| 3  *No change* | 5353.43 | 5321.25 | .11 | .13 | .00 | 0.65 |
| 3  *Linear* | 5350.93 | 5308.02 | .30 | .31 | .00 | 0.76 |
| 3  *Nonlinear* | 5364.33 | 5310.69 | .58 | .59 | .00 | 0.73 |

*Note.* The bolded two-group model showed the best model fit.

**Justification of the Choices of the Model for Positive Attentional Bias**

Although the 3-class linear model had the lowest BIC and AIC values, it had non-significant p values for the VLMR test and the Adj. LMR test (*p* = .30 and .31, respectively), suggesting that the 3-class model was not significantly better than the 2-class model. Among the 2-class models, the 2-class linear model had the lowest BIC and AIC values compared to the 2-class non-change and nonlinear models. The 2-class linear model also had significant p values for the VLMR test, Adj. LMR test and bootstrap likelihood ratio test (BLRT), suggesting that it was significantly better than the general 1-class model. Additionally, it had higher entropy (0.68) compared to the 2-class non-change model (0.64). BIC, LMR and BLRT have been recommended as reliable indices for model selection in latent class growth analysis (Jung & Wickrama, 2008). Thus, the 2-class linear model was selected as the best model for the unconditional trajectories of positive attentional bias.

**Table A2.3**

*Fit Statistics for Latent Class Growth Analysis Models Representing One to Four Trajectory Groups of Internalizing Symptoms (Unconditional)*

| Group number  *Shape* | BIC | AIC | VLMR  (p Value) | Adj. LMR  (p Value) | BLRT  (p Value) | Entropy |
| --- | --- | --- | --- | --- | --- | --- |
| 1  *No change* | 9488.36 | 9466.91 | NA | NA | NA | NA |
| 1  *Linear* | 9470.18 | 9438.00 | NA | NA | NA | NA |
| 1  *Nonlinear* | 9772.81 | 9747.78 | NA | NA | NA | NA |
| 2  *No change* | 9427.41 | 9398.80 | .00 | .00 | .00 | 0.91 |
| 2  *Linear* | 9390.42 | 9347.51 | .01 | .01 | .00 | 0.94 |
| 2  *Nonlinear* | 9379.34 | 9340.00 | .00 | .00 | .00 | 0.96 |
| 3  *No change* | 9427.73 | 9391.97 | .10 | .12 | .00 | 0.91 |
| 3  *Linear* | 9380.92 | 9327.29 | .37 | .38 | .00 | 0.91 |
| 3  *Nonlinear* | **9294.79** | **9241.15** | **.01** | **.01** | **.00** | **0.94** |
| 4  *No change* | 9428.09 | 9385.18 | .62 | .65 | .00 | 0.80 |
| 4  *Linear* | 9372.75 | 9308.39 | .28 | .29 | .00 | 0.90 |
| 4  *Nonlinear* | 9280.03 | 9212.08 | .31 | .32 | .00 | 0.96 |

*Note.* The bolded three-group model showed the best model fit.

**Justification of the Model Choice for Internalizing Symptoms**

Although the 4-class nonlinear model had the lowest AIC (9212.08) and BIC (9280.03) values, the 3-class nonlinear model was selected based on several considerations. The 3-class nonlinear model showed significant improvement over the 2-class model, with significant p values for VLMR (p = .01), Adj. LMR (p = .01), and BLRT (p < .001). In contrast, the 4-class model showed non-significant VLMR and Adj. LMR tests (p = .31 and .32), suggesting that the additional class did not provide significant improvement over the 3-class solution. The 3-class nonlinear model also demonstrated good classification quality with an entropy of 0.94. Additionally, the 3-class solution provides a more parsimonious and interpretable representation of internalizing symptom trajectories during this developmental period.

**A3. Statistical Analyses Comparing Participants who Dropped out with Those Who Completed all the Assessments**

Children who dropped out or did not complete all of the assessments (N=58) were not significantly different from children who completed all four assessments in terms of scores in anxiety and depression symptoms, t (320)=.46, p=.65, negative attentional bias, t (316)=.82, p=.41, positive attentional bias, t (317)=-1.17, p=.24, active coping style, t (320)=-.76, p=.45, and avoidant coping style, t (320)=-.59, p=.55, at time one.

**A4. Scales to Measure Attentional Bias, Anxiety and Depression Symptoms, and Active and Avoidant Coping Styles**

**Table A4.1***The Full Scale of the Chinese Versions of the APNIS*

| 答案没有正确好坏之分，请真实地填写  以下的句子能够正确地形容你吗？  There are no right or wrong, good or bad answers. Please answer honestly.  To what extent do the following statements accurately describe you? | | 非常不正确Very untrue | 有点不正确Somewhat untrue | 中立Neutral | 有点正确Somewhat true | 非常正确Very true |
| --- | --- | --- | --- | --- | --- | --- |
|  | 我会留意他人所做的正面的事情。I notice the positive things others do. | 1 | 2 | 3 | 4 | 5 |
|  | 我不能忘记自己在某些事情上表现差劲的时候。 I can’t forget times when I did poorly at something. | 1 | 2 | 3 | 4 | 5 |
|  | 我不会忘记别人伤害我的事。I don’t forget when people hurt me. | 1 | 2 | 3 | 4 | 5 |
|  | 我特别留意在电视新闻上播出的坏新闻。I pay special attention to bad news on TV. | 1 | 2 | 3 | 4 | 5 |
|  | 那些我做得不好的事情总是缠绕在我的脑海中。The things I did poorly dwell on my mind. | 1 | 2 | 3 | 4 | 5 |
|  | 我对生活上的许多小小的快乐非常敏感。I’m sensitive to many small pleasures in life. | 1 | 2 | 3 | 4 | 5 |
|  | 我注意那些令自己快乐的事情。I notice things that make me happy. | 1 | 2 | 3 | 4 | 5 |
|  | 我可以迅速地注意到别人的过失。I quickly notice other people’s mistakes. | 1 | 2 | 3 | 4 | 5 |
|  | 生命中有很多事物都是我所喜欢的。There are many things in life I enjoy. | 1 | 2 | 3 | 4 | 5 |
|  | 对我来说留意到自己的不足之处是重要的。It’s important for me to notice my shortcomings. | 1 | 2 | 3 | 4 | 5 |
|  | 我特别能留意到我的那些被家人和朋友称赞的特点。I especially notice qualities about myself that my family and friends praise. | 1 | 2 | 3 | 4 | 5 |
|  | 当有不对劲的事情发生时，即使是件很琐碎的事，我也能注意到。I notice when something is wrong, even if it’s trivial. | 1 | 2 | 3 | 4 | 5 |
|  | 我留意到自己的优点。I notice my own strengths. | 1 | 2 | 3 | 4 | 5 |
|  | 对我来说，记着别人的好是重要的。It’s important for me to remember the good in others. | 1 | 2 | 3 | 4 | 5 |
|  | 我想在多方面改善自己。I want to better myself in many ways. | 1 | 2 | 3 | 4 | 5 |
|  | 无论谁在微笑，我都会留意到那张快乐的脸孔。I notice happy faces, whoever is smiling. | 1 | 2 | 3 | 4 | 5 |
|  | 我能察觉到顺心如意的时候。I can detect when things are going well. | 1 | 2 | 3 | 4 | 5 |
|  | 我特别留意自己做得成功的事情。I pay special attention to things I’ve done successfully. | 1 | 2 | 3 | 4 | 5 |
|  | 别人批评我时，他们的意见会在我的脑海中逗留一段时间。When criticized, others’ opinions stay in my mind for a while. | 1 | 2 | 3 | 4 | 5 |
|  | 无论参加任何活动，我都可以轻易看到活动有趣的一面。I can easily see the fun side of any activity I join. | 1 | 2 | 3 | 4 | 5 |
|  | 我在完成任务时，总会注意到当中的困难。When completing tasks, I always notice the difficulties. | 1 | 2 | 3 | 4 | 5 |
|  | 我习惯注意以前那些令自己不快乐的情境。I tend to dwell on past situations that made me unhappy. | 1 | 2 | 3 | 4 | 5 |
|  | 我的记忆里，大多是自己快乐的时光。My memories are mostly of happy times. | 1 | 2 | 3 | 4 | 5 |
|  | 我喜欢称赞别人的优点。I like to praise others’ strengths. | 1 | 2 | 3 | 4 | 5 |
|  | 我担心坏事情会发生在我身上。I worry bad things will happen to me. | 1 | 2 | 3 | 4 | 5 |
|  | 如果我发现别人有需要改善的地方，我会告诉他。If I notice areas where others could improve, I point it out to them. | 1 | 2 | 3 | 4 | 5 |
|  | 我通常认为好的事情会发生在我身上。I generally believe good things will happen to me. | 1 | 2 | 3 | 4 | 5 |
|  | 我通常认为好的事情会发生在我身边的人身上。I generally believe good things will happen to people around me. | 1 | 2 | 3 | 4 | 5 |
|  | 我觉得自己经常犯错。I feel I make mistakes often. | 1 | 2 | 3 | 4 | 5 |
|  | 有时我认为其他人想欺骗我。Sometimes I think others want to deceive me. | 1 | 2 | 3 | 4 | 5 |

|  | 我尝试努力地去达到自己的个人目标。I strive hard to achieve my personal goals. | 1 | 2 | 3 | 4 | 5 |
| --- | --- | --- | --- | --- | --- | --- |
|  | 我认为其他人帮助了我许多。I believe others have helped me a lot. | 1 | 2 | 3 | 4 | 5 |
|  | 有许多人都是我不喜欢的。There are many people I dislike. | 1 | 2 | 3 | 4 | 5 |
|  | 我对自己的未来感到乐观。I feel optimistic about my future. | 1 | 2 | 3 | 4 | 5 |
|  | 我认为每个人都有些好的品质。I believe everyone has some good qualities. | 1 | 2 | 3 | 4 | 5 |
|  | 我经常挑剔别人。I often find fault with others. | 1 | 2 | 3 | 4 | 5 |
|  | 能一直做自己喜欢的事是很好的。It’s great to always do things I enjoy. | 1 | 2 | 3 | 4 | 5 |
|  | 我总觉得别人看上去是快乐的。I always feel others look happy. | 1 | 2 | 3 | 4 | 5 |
|  | 我担心一些不幸的事情会发生在我身边的人身上。I worry unfortunate things will happen to people close to me. | 1 | 2 | 3 | 4 | 5 |
|  | 对我来说，对别人有好感是重要的。It’s important for me to think well of others. | 1 | 2 | 3 | 4 | 5 |

The Chinese version of Revised Child and Adolescent Disorder Scale was downloaded from <http://www.childfirst.ucla.edu/Resources.html.>

**Table A4.2***English Translation of the Active and Avoidance Coping Scale for Chinese Children*

| Recalling the past few months, when you encountered difficulties brought by COVID-19, did you have the following reactions?回忆过去几个月里，你在遇到新冠肺炎带來的困难时，是否会有以下的反应？ | 从来没有  Never | 有时  Sometimes | 经常  Often | 总是  Always |
| --- | --- | --- | --- | --- |
| - 在我遇到困难的时候，我在行动前想过如何解决它。When I encountered difficulties, I thought about how to solve them before taking action. | 1 | 2 | 3 | 4 |
| - 在我遇到困难的时候，我不理它。When I encountered difficulties, I ignored them. | 1 | 2 | 3 | 4 |
| - 在我遇到困难的时候，我逃避它。When I encountered difficulties, I avoided them. | 1 | 2 | 3 | 4 |
| - 在我遇到困难的时候，我为了让情况变好而做了一些事情。When I encountered difficulties, I did something to make the situation better. | 1 | 2 | 3 | 4 |
| - 在我遇到困难的时候，我告诉自己事情会好起来的。When I encountered difficulties, I told myself that things would get better. | 1 | 2 | 3 | 4 |
| - 在我遇到困难的时候，我提醒自己我的情況比其他很多孩子更好。When I encountered difficulties, I reminded myself that my situation was better than that of many other children. | 1 | 2 | 3 | 4 |
| - 在我遇到困难的时候，我幻想着一切都很好。When I encountered difficulties, I fantasized that everything was fine. | 1 | 2 | 3 | 4 |
| - 在我遇到困难的时候，我试着把它抛到脑后。When I encountered difficulties, I tried to put it out of my mind. | 1 | 2 | 3 | 4 |
| - 在我遇到困难的时候，我在决定做什么之前考虑过这样做的后果。When I encountered difficulties, I considered the consequences before deciding what to do. | 1 | 2 | 3 | 4 |
| - 在我遇到困难的时候，我告诉自己它会好转的。When I encountered difficulties, I told myself it would improve. | 1 | 2 | 3 | 4 |
| - 在我遇到困难的时候，我告诉自己我能处理它。When I encountered difficulties, I told myself I could handle it. | 1 | 2 | 3 | 4 |
| - 在我遇到困难的时候，我远离那些让我感到不愉快的事情。When I encountered difficulties, I stayed away from things that made me feel unpleasant. | 1 | 2 | 3 | 4 |
| - 在我遇到困难的时候，我通过改变我的做法来让情况变得更好。When I encountered difficulties, I made things better by changing my approach. | 1 | 2 | 3 | 4 |
| - 在我遇到困难的时候，我告诉自己我过去有处理过它。When I encountered difficulties, I told myself I had handled it before. | 1 | 2 | 3 | 4 |
| - 在我遇到困难的时候，我想过它为什么会发生。When I encountered difficulties, I thought about why it happened. | 1 | 2 | 3 | 4 |
| - 在我遇到困难的时候，我不去想它。When I encountered difficulties, I didn't think about it. | 1 | 2 | 3 | 4 |
| - 在我遇到困难的时候，我告诉自己不管发生什么我都能处理。When I encountered difficulties, I told myself I could handle whatever happened. | 1 | 2 | 3 | 4 |
| - 在我遇到困难的时候，我告诉自己，从长远来看，事情会朝着最好的方向发展。When I encountered difficulties, I told myself that in the long run, things would work out for the best. | 1 | 2 | 3 | 4 |
| - 在我遇到困难的时候，我幻想它向我希望的方向发展。When I encountered difficulties, I fantasized about it turning out the way I hoped. | 1 | 2 | 3 | 4 |
| - 在我遇到困难的时候，我提醒自己我知道该怎么做。When I encountered difficulties, I reminded myself I knew what to do. | 1 | 2 | 3 | 4 |
| 1. 在我遇到困难的时候，我想过哪些是处理它的最好方法。When I encountered difficulties, I thought about the best ways to handle it. | 1 | 2 | 3 | 4 |
| 1. 在我遇到困难的时候，我就直接忘掉它。When I encountered difficulties, I simply forgot about it. | 1 | 2 | 3 | 4 |
| 在我遇到困难的时候，我告诉自己它会解决的。  When I encountered difficulties, I told myself it would work out. | 1 | 2 | 3 | 4 |
| 在我遇到困难的时候，我避开那些让我感觉不愉快的人。  When I encountered difficulties, I avoided people who made me feel unpleasant. | 1 | 2 | 3 | 4 |
| 在我遇到困难的时候，我提醒自己，总体上对我来说，情況很好。When I encountered difficulties, I reminded myself that overall, things were good for me. | 1 | 2 | 3 | 4 |
| 在我遇到困难的时候，我为了解决它而做了一些事情。When I encountered difficulties, I did something to solve it. | 1 | 2 | 3 | 4 |
| 在我遇到困难的时候，我通过好好思考来理解它。When I encountered difficulties, I tried to understand it by thinking it through carefully. | 1 | 2 | 3 | 4 |
| 在我遇到困难的时候，我提醒自己我有哪些优势。When I encountered difficulties, I reminded myself of my strengths. | 1 | 2 | 3 | 4 |
| 在我遇到困难的时候，我祈求糟糕的事情不会发生。When I encountered difficulties, I prayed that bad things wouldn't happen | 1 | 2 | 3 | 4 |
| 在我遇到困难的时候，我思考我需要了解什么才能解决它。When I encountered difficulties, I thought about what I needed to understand to solve it. | 1 | 2 | 3 | 4 |
| 在我遇到困难的时候，我为了逃避它去到房间里。When I encountered difficulties, I went to another room to escape it. | 1 | 2 | 3 | 4 |
| 在我遇到困难的时候，我为了从中得到最大的好处而做了一些事情。When I encountered difficulties, I did something to make the best of it. | 1 | 2 | 3 | 4 |
| 在我遇到困难的时候，我思考我能从中学到什么。When I encountered difficulties, I thought about what I could learn from it. | 1 | 2 | 3 | 4 |
| 在我遇到困难的时候，我祈求情况可以更好。When I encountered difficulties, I prayed that the situation could be better. | 1 | 2 | 3 | 4 |
| 在我遇到困难的时候，我尝试弄明白它发生的原因。When I encountered difficulties, I tried to figure out the reason it happened | 1 | 2 | 3 | 4 |

**A5 Translation Procedure of the Children’s Coping Strategies Checklist – Revised1 (CCSC-R1)**

The active coping subscale and the avoidant coping subscale of the Children’s Coping Strategies Checklist – Revised1 (CCSC-R1) were used to measure active and avoidant coping styles (Program for Prevention Research, 1999). Because the scale doesn’t have a Chinese version, the CCSC-R1 scale was translated into Chinese. The translation process was guided by the Guidelines for translating and adapting psychological instruments (Gudmundsson, 2009). Translators fluent in both English and Chinese were selected from postgraduate students majoring in psychology. Translation process included forward translation, back translation, and pilot testing of the preliminary version. First, the revised Children’s Coping Strategies Checklist—active and avoidant coping subscale was translated into Chinese by two independent PhD students in psychology and a bilingual independent PhD student majoring in social work. Then, the initial Chinese translation was blind back-translated by another independent bilingual student in Master of psychology program. Finally, all translators and a clinical psychology professor who had experience in the translation of psychological instruments had discussion on the words and sentences to resolve any discrepancies and biases in the translation. As a result of the discussion, the preliminary Chinese version of the scale was decided and was later administered to a small group of 50 children for pilot testing. Any difficulty in understanding the Chinese items during the pilot testing was resolved, and the final version of the Chinese translation of active and avoidant coping scales from CCSC-R was created.

**A6. Exploratory Factor Analysis and Confirmatory Factor Analysis**

**Exploratory Factor Analysis**

Exploratory factor analysis (EFA) was conducted to explore the factor structure of the APNIS with SPSS version 22 with an independent sample of 120 children (age from 11 to 13 years). First, the Kaiser–Meyer–Olkin (KMO) test was performed. KMO value was .773, and Bartlett’s test of Sphericity was significant, *χ2*= 2187.350, *df*=780, *p*< .001, suggesting good factorability. This suggested that Principle Component Analysis (PCA) is suitable to explore the factor structure of the scale. Thus, PCA with Varimax rotation was conducted. The first PCA revealed 10 factors, which accumulatively accounted for 66.54% of the variance. 20 items had cross-loadings with a difference below .30, which were deleted. The second PCA displayed that 4 items had cross-loadings, which were also deleted. The third PCA showed that another four items had cross-loadings. These items were subsequently deleted. The fourth PCA showed that four items were loaded on factors that had fewer than three items. Thus, these factors were deleted. The final PCA showed a clear two-factor structure that had eigenvalues above 1. The first factor was defined as “positive attentional bias”. It included items 13, 17, 18, and 40, accounting for 28.226% of the variance. The second factor was named “negative attentional bias” which included item 2, item 4, item 5, and item 22. This factor explained 36.501% of the variance. The two-factor model explained 64.726% of the variance.

**Confirmatory Factor Analysis**

We conducted CFA to confirm the two-factor structure of APNIS with the sample of 264 children using AMOS 24 (Arbuckle, 2014). The fit indices revealed that the structural validity of the two-factor 8-item brief APNIS for children was good (RMSEA =.065; GFI=.963, AGFI=.934; CFI=.935, TLI=.909) . Although the Chi-Square Test of Model Fit was significant, *χ^2^* (20) = 42.545, *p*<.005, this index tends to be significant in a large sample (Schumacker & Lomax, 2010).

**Table A6.1***Factor Loadings for Exploratory Factor Analysis With Varimax Rotation of the APNIS for the Chinese Children Sample (n=111).*

| Items | Factor 1 | Factor 2 |
| --- | --- | --- |
| Factor 1: Negative attentional bias |  |  |
| Item 2 I can’t forget the times I have performed poorly at something. 我无法忘记自己在某些事情上表现不好的时候。 | .823 |  |
| Item 4 I am particularly aware of the bad news that  appears in TV news broadcasts我会特别注意电视新闻上  的坏消息 | .787 |  |
| Item 5 Things that I am not good at are always on my mind那些我做得不好的事情总是萦绕在我的脑海中。 | .873 |  |
| Item 22 I usually notice situations that made me feel bad in the past我经常注意过去那些令自己不快乐的情境。 | .826 |  |
| Factor 2: Positive attentional bias |  |  |
| Item 13 I pay attention to my positive characteristics of myself. 我会注意到自己的正面特质。  Item 17 I realize and pay attention to moments when everything is going well. 我会察觉并注意到事事顺利的时刻。 |  | .814  .879 |
| Item 18 I am particularly aware of things that I am  successful at. 我特别留意自己做得成功的事情。  Item 40 It is important for me to remember the good  things about others. 对我来说, 认为别人好很重要。 |  | .873  .381 |
|  |  |  |

**A7. LCGA with Measurement Models for Positive Attentional Bias, Negative Attentional Bias, and Internalizing Symptoms**

**Positive Attentional Bias**

***Conditional Model***

After including gender, active and avoidant coping styles, anxiety symptoms and depression symptoms at T1 in the model, LCGA which included the measurement model for positive attentional bias showed much poorer model fit than the trajectory of positive attentional bias that did not include the measurement model. Refer to the Table A7.1 below.

**Table A7.1**

*Model Fit for the Conditional Trajectories of Positive Attentional Bias with Measurement Model*

| **Group number** | **BIC** | **AIC** | **VLMR**  **(p Value)** | **Adj. LMR (p Value)** | **BLRT**  **(p Value)** | **Entropy** |
| --- | --- | --- | --- | --- | --- | --- |
| 1  Nonchange | 12805.99 | 12645.33 | NA | NA | NA | NA |
| 1  Linear | unidentified | unidentified | unidentified | unidentified | unidentified | unidentified |
| 1  Nonlinear | unidentified | unidentified | unidentified | unidentified | unidentified | unidentified |
| 2 Nonchange | 12739.24 | 12561.85 | 0.56 | 0.56 | 1.00 | 0.99 |
| 2  Linear | 12714.96 | 12524.18 | 0.40 | 0.40 | 0.00 | 0.77 |
| 2 Nonlinear | 12716.19 | 12542.14 | 0.19 | 0.21 | 0.00 | 0.70 |
| 3  Nonchange | 12721.67 | 12534.23 | 0.24 | 0.24 | 0.00 | 0.70 |
| 3 Linear | 12713.13 | 12529.03 | 0.42 | 0.42 | 0.00 | 0.66 |
| 3 Nonlinear | 12730.01 | 12502.41 | 0.72 | 0.72 | 1.00 | 0.68 |

***Unconditional Model***

For unconditional model without including gender, active and avoidant coping styles, anxiety symptoms and depression symptoms at T1 in the model, LCGA which included the measurement model for positive attentional bias showed much poorer model fit than the trajectory of positive attentional bias that did not include the measurement model. Refer to the Table A7.2 below.

**Table A7.2***Model Fit for the Unconditional Trajectories of Positive Attentional Bias with Measurement Model*

| **Group number** | **BIC** | **AIC** | **VLMR**  **(p Value)** | **Adj. LMR (p Value)** | **BLRT**  **(p Value)** | **Entropy** |
| --- | --- | --- | --- | --- | --- | --- |
| 1  Nonchange | 12805.99 | 12645.33 | NA | NA | NA | NA |
| 1  Linear | unidentified | unidentified | unidentified | unidentified | unidentified | unidentified |
| 1  Nonlinear | unidentified | unidentified | unidentified | unidentified | unidentified | unidentified |
| 2 Nonchange | 12739.24 | 12561.85 | 0.56 | 0.56 | 1.00 | 0.99 |
| 2  Linear | 12716.19 | 12542.14 | 0.19 | 0.21 | .00 | 0.70 |
| 2 Nonlinear | 12710.20 | 12542.85 | 0.12 | 0.14 | .00 | 0.67 |
| 3  Nonchange | 12721.67 | 12534.23 | 0.30 | 0.32 | .00 | 0.63 |
| 3  Linear | 12713.12 | 12529.03 | 0.42 | 0.42 | .00 | 0.66 |
| 3 Nonlinear | 12728.15 | 12534.01 | .43 | .43 | .00 | 0.66 |

**Negative Attentional Bias**

***Conditional Model***

After including gender, active and avoidant coping styles, anxiety and depression symptoms at T1 in the model, LCGA which included the measurement model for negative attentional bias showed much poorer model fit than the trajectory of negative attentional bias that did not include the measurement model. Refer to the Table 7.3 below.

**Table A7.3**

*Model Fit for the Conditional Trajectories of Negative Attentional Bias with Measurement Model*

| **Group number** | **BIC** | **AIC** | **VLMR**  **(p Value)** | **Adj. LMR (p Value)** | **BLRT**  **(p Value)** | **Entropy** |
| --- | --- | --- | --- | --- | --- | --- |
| 1  Nonchange | 11124.33 | 10963.67 | NA | NA | NA | NA |
| 1  Linear | unidentified | unidentified | unidentified | unidentified | unidentified | unidentified |
| 1  Nonlinear | unidentified | unidentified | unidentified | unidentified | unidentified | unidentified |
| 2 Nonchange | 10907.73 | 10723.64 | 0.00 | 0.00 | 0.00 | 0.77 |
| 2  Linear | 11172.46 | 10981.67 | 0.50 | 0.50 | 1.00 | 0.00 |
| 2 Nonlinear | 12716.19 | 12542.14 | 0.19 | 0.21 | 0.00 | 0.70 |
| 3  Nonchange | 11199.19 | 10991.67 | 1.00 | 1.00 | 1.00 | 0.00 |
| 3 Linear | 12713.13 | 12529.03 | 0.42 | 0.42 | 0.00 | 0.66 |
| 3 Nonlinear | 12730.01 | 12502.41 | 0.72 | 0.72 | 1.00 | 0.68 |

***Unconditional Model***

For unconditional model without including gender, active and avoidant coping styles, anxiety symptoms and depression symptoms at T1 in the model, LCGA which included the measurement model for negative attentional bias showed much poorer model fit than the trajectory of negative attentional bias that did not include the measurement model. Refer to the Table A7.4 below.

**Table A7.4***Model Fit for the Unconditional Trajectories of Negative Attentional Bias with Measurement Model*

| **Group number** | **BIC** | **AIC** | **VLMR**  **(p Value)** | **Adj. LMR (p Value)** | **BLRT**  **(p Value)** | **Entropy** |
| --- | --- | --- | --- | --- | --- | --- |
| 1  Nonchange | 12805.99 | 12645.33 | NA | NA | NA | NA |
| 1  Linear | unidentified | unidentified | unidentified | unidentified | unidentified | unidentified |
| 1  Nonlinear | unidentified | unidentified | unidentified | unidentified | unidentified | unidentified |
| 2 Nonchange | 12739.24 | 12561.85 | 0.56 | 0.56 | 1.00 | 0.99 |
| 2  Linear | 12716.19 | 12542.14 | 0.19 | 0.21 | .00 | 0.70 |
| 2 Nonlinear | unidentified | unidentified | unidentified | unidentified | unidentified | unidentified |
| 3  Nonchange | 12721.67 | 12534.23 | 0.30 | 0.32 | .00 | 0.63 |
| 3  Linear | 10879.13 | 10661.57 | 0.56 | 0.56 | .00 | 0.81 |
| 3 Nonlinear | unidentified | unidentified | unidentified | unidentified | unidentified | unidentified |

**Internalizing Symptoms**

***Conditional Model***

After including gender, active and avoidant coping styles, negative attentional bias and positive attentional bias at T1 in the model, LCGA which included the measurement model for internalizing symptoms showed insignificant VLMR, Adj.LMR, or BLRT values, suggesting poorer model fit than the trajectory of internalizing symptoms that did not include the measurement model. Refer to the Table 7.5 below.

**Table A7.5**

*Model Fit for the Conditional Trajectories of Internalizing Symptoms with Measurement Model*

| **Group number** | **BIC** | **AIC** | **VLMR**  **(p Value)** | **Adj. LMR (p Value)** | **BLRT**  **(p Value)** | **Entropy** |
| --- | --- | --- | --- | --- | --- | --- |
| 1  Nonchange | 9916.81 | 9795.23 | NA | NA | NA | NA |
| 1  Linear | 9922.39 | 9797.23 | NA | NA | NA | NA |
| 1  Nonlinear | 9927.96 | 9799.23 | NA | NA | NA | NA |
| 2 Nonchange | 6226.43 | 6116.65 | .24 | .24 | .00 | 0.69 |
| 2  Linear | unidentified | unidentified | unidentified | unidentified | unidentified | unidentified |
| 2 Nonlinear | 6245.71 | 6121.77 | .06 | .07 | .00 | 0.69 |
| 3  Nonchange | unidentified | unidentified | unidentified | unidentified | unidentified | unidentified |
| 3 Linear | 6264.06 | 6118.87 | .21 | .21 | .31 | 0.80 |
| 3 Nonlinear | unidentified | unidentified | unidentified | unidentified | unidentified | unidentified |

***Unconditional Model***

For unconditional model without including gender, active and avoidant coping styles, negative attentional bias and positive attentional bias at T1 in the model, LCGA which included the measurement model for internalizing symptoms showed insignificant VLMR, Adj.LMR, and BLRT values, indicating much poorer model fit than the trajectory of internalizing symptoms that did not include the measurement model. Refer to the Table A7.6 below.

**Table A7.6***Model Fit for the Unconditional Trajectories of Internalizing Symptoms with Measurement Model*

| Group number | BIC | AIC | VLMR  (p Value) | Adj. LMR (p Value) | BLRT  (p Value) | Entropy |
| --- | --- | --- | --- | --- | --- | --- |
| 1  Nonchange | 6568.23 | 6482.41 | NA | NA | NA | NA |
| 1  Linear | 6573.81 | 6484.41 | NA | NA | NA | NA |
| 1  Nonlinear | 6579.38 | 6486.41 | NA | NA | NA | NA |
| 2 Nonchange | unidentified | unidentified | unidentified | unidentified | unidentified | unidentified |
| 2  Linear | 6377.64 | 6517.38 | unidentified | unidentified | unidentified | 0.79 |
| 2 Nonlinear | 6601.69 | 6494.41 | .19 | .19 | 1.00 | 0.00 |
| 3  Nonchange | unidentified | unidentified | unidentified | unidentified | unidentified | unidentified |
| 3  Linear | 6505.25 | 6394.40 | .50 | .50 | 1.00 | .87 |
| 3 Nonlinear | 6623.99 | 6502.41 | .24 | .24 | 1.00 | 1.00 |

In conclusion, LCGA with measurement models has poorer model fit indices. Thus, using measurement models in LCGA would produce less accurate results of trajectory groups, which would negatively impact the findings of subsequent analyses.

**A8.** **Preliminary Analyses of Longitudinal Measurement Invariance**

**Configural Invariance**

The configural model for positive attentional bias across 4 time points showed a good overall fit to the data: CFI=0.965; TLI= 0.937; RMSEA=0.039; SRMR=0.056. These suggest that the measurement for positive attentional bias had good configural measurement invariance across 4 time points.

The configural model for negative attentional bias across 4 time points showed a good overall fit to the data: CFI=0.965; TLI= 0.944; RMSEA=0.039; SRMR=0.045. These indicate that the measurement for negative attentional bias had good configural measurement invariance across 4 time points.

The configural model for internalizing symptoms across 4 time points had good model fit indices: CFI=0.935; TLI= 0.923; RMSEA=0.081; SRMR=0.034. These indicate that the measurement for internalizing symptoms had good configural measurement invariance across 4 time points.

**Metric and Scalar Invariance**

Longitudinal measurement invariance was examined for the main study measures (API, ANI, RCADS) across four time points. As shown in Table A8.1, changes in model fit indices (ΔCFI, , ΔRMSEA) were compared between configural, metric, and scalar models. According to Chen (2007), changes in CFI<.01 or in RMSEA<.015 indicate metric and scalar longitudinal invariance. For **API** (Attention to Positive Information) and **ANI (**Attention to Negative Information), all changes in CFI, and RMSEA were within the recommended thresholds, supporting metric and scalar invariance. For **RCADS** (Revised Child Anxiety and Depression Scale), longitudinal invariance at the scalar level was supported with changes in RMSEA< .015 (Chen, 2007).

**Table A8.1**

*Longitudinal Invariance and Changes in Model Fit Indices for the Major Measures.*

| Measure | Model | ΔCFI | ΔRMSEA | Hold |
| --- | --- | --- | --- | --- |
| API | Configural | - | - | Yes |
| API | Metric | .005 | -.005 | Yes |
| API | Scalar | -.007 | -.003 | Yes |
| ANI | Configural | - | - | Yes |
| ANI | Metric | .001 | -.004 | Yes |
| ANI | Scalar | -.008 | -.002 | Yes |
| RCADS | Configural | - | - | Yes |
| RCADS | Metric | -.003 | < .001 | Yes |
| RCADS | Scalar | -.031 | < .015 | Yes |

RCADS = Revised Child Anxiety and Depression Scale. API = Attention to Positive Information Scale. ANI = Attentional to Negative Information Scale.

**A9. Gender Differences in the Trajectories**

**Negative Attentional Bias**

Results from latent class growth trajectory (conditional model) also showed that there was no significant gender difference in the probabilities of displaying negative attentional bias trajectories. Compared to the low negative attentional bias trajectory membership, boys and girls did not significantly differ in displaying the moderate negative attentional bias trajectory membership, b = -0.131, *p* = 0.771, and the high negative attentional bias trajectory membership, b = -0.912, *p* = 0.115.

**Positive Attentional Bias**

Results from latent class growth trajectory (conditional model)also showed that there was no significant gender difference in the probabilities of displaying a positive attentional bias trajectory. Compared to the low positive attentional bias trajectory membership, boys and girls did not significantly differ in displaying the high positive attentional bias trajectory membership, b =0.316 , *p* = 0.444.

**Internalizing Symptoms**

Results from latent class growth trajectory (conditional model) also showed that there was no significant gender difference in the probabilities of displaying a developmental trajectory of internalizing symptoms. Compared to the low internalizing trajectory membership, boys and girls did not significantly differ in displaying the moderate internalizing trajectory membership, b = -0.836, p = 0.081, and the high internalizing trajectory membership, b =-0.020 , p = 0.969.

**References**

Arbuckle, J. L. (2014). Amos (Version 23.0)[Computer Program]. Chicago: IBM SPSS.

Chen, F. F. (2007). Sensitivity of Goodness of Fit Indexes to Lack of Measurement Invariance. *Structural Equation Modeling, 14*(3), 464-504. doi:10.1080/10705510701301834

Gudmundsson, E. (2009). Guidelines for translating and adapting psychological instruments. *Nordic Psychology, 61*(2), 29-45. doi:<http://dx.doi.org/10.1027/1901-2276.61.2.29>

Jung, T., & Wickrama, K. A. S. (2008). An Introduction to Latent Class Growth Analysis and Growth Mixture Modeling. *Social and personality psychology compass, 2*(1), 302-317. doi:10.1111/j.1751-9004.2007.00054.x

Nguena Nguefack, H. L., Pagé, M. G., Choinière, M., & Lacasse, A. (2019). Trajectory Modelling Techniques Useful to Pain Research: A Narrative Comparison Of Approaches: Research poster abstract. *Canadian journal of pain*. doi:10.1080/24740527.2019.1591798

Research, P. f. P. (1999). *Manual for the Children's Coping Strategies Checklist and the How I Coped Under Pressure Scale* Temp, AZ: Arizona State University.

Schumacker, R. E., & Lomax, R. G. (2010). *A beginner's guide to structural equation modeling* (3rd ed. ed.): Routledge/Taylor & Francis Group, New York, NY.
